# Efficacy and Safety of Aspirin, Promethazine, and Micronutrients for Rapid Clinical Recovery in Mild to Moderate COVID-19 Patients: A Randomized Controlled Clinical Trial

**DOI:** 10.7759/cureus.25467

**Published:** 2022-05-30

**Authors:** G. Sunil Kumar, Atul Vadgaonkar, Srilata Purunaik, Rohit Shelatkar, Vidyadhar G Vaidya, Gayatri Ganu, Aditya Vadgaonkar, Shashank Joshi

**Affiliations:** 1 Cardiology, S K Clinic & Scans, Thiruvanathapuram, IND; 2 General Medicine, Vijay Nursing Home, Nashik, IND; 3 Respiratory Medicine, Swasthi Orthopaedic and Respiratory Health Care, Bengaluru, IND; 4 Pharmacology and Therapeutics, Vitabiotics Ltd., London, GBR; 5 General Surgery, Lokmanya Medical Research Centre, Pune, IND; 6 Pharmacology and Therapeutics, Mprex Healthcare Pvt. Ltd., Pune, IND; 7 Medicine, King Edward Memorial Hospital and Seth Gordhandas Sunderdas Medical College, Mumbai, IND; 8 Endocrinology, Lilavati Hospital, Mumbai, IND

**Keywords:** covid-19, vitamin d, selenium, zinc, promethazine, vitamin c, aspirin

## Abstract

Introduction

In the present study, the combination of two tablets, one with Aspirin and Promethazine and the other with vitamin D3, C, and B3 along with zinc and selenium supplementation was proposed as an intervention (APMV2020). The ingredients in the formulation represent a precise, tailored therapy for the symptoms of COVID-19, combined with natural constituents to help the body itself build immunity to recover from infection. The present study was conducted to clinically validate the safety and efficacy of the APMV2020 tablets.

Trial design

The present trial is a randomized, multicentric, controlled clinical trial involving 260 mild to moderate COVID-19 patients. The treatment duration was of 10 days.

Methodology

The subjects were randomized to receive either the control intervention (clinical management protocol for COVID-19 advocated by the Indian Council of Medical Research (ICMR) or the test intervention (treatment with APMV2020 tablets along with the standard control treatment. The assessment days were baseline, days five and 10.

Results

APMV2020 significantly (<0.05) improved symptoms of COVID-19 like cough, myalgia, headache, and anosmia as compared to the control group. APMV2020 treatment also reduced inflammatory markers like lactate dehydrogenase (LDH), ferritin, and C-reactive protein (CRP).

Conclusion

APMV2020 can prove as a good candidate to be integrated into the COVID-19 management protocol. As it can offer speedy clinical recovery to reduce the burden on healthcare infrastructure, second, the combination shows significant anti-inflammatory potential to improve prognosis, and lastly, the immunomodulatory properties offer long-term protection that can help in combating long COVID symptoms and complications.

## Introduction

Severe acute respiratory syndrome coronavirus 2 (SARS-CoV-2) infection presents itself clinically from asymptomatic, to mild to moderate respiratory and non-respiratory symptoms, to severe COVID-19 pneumonia and acute respiratory distress syndrome (ARDS) with multiorgan failure. There is worldwide emergence of various long-lasting complications after SARS-CoV-2 infection (the post-COVID syndrome or long COVID). The SARS-Cov-2 infection leads to a host response that triggers wide-ranging immuno-inflammatory, thrombotic, and parenchymal derangements in COVID 19 [1].

The virus mutations led to several phases or waves of COVID 19. The cross-reactivity on one hand and the viral mutations, on the other hand, explain the evolution of the pandemic until the summer of 2020 [2]. The primary mutations observed during the summer of 2021 appeared in the spike protein [3], which has noticeably increased its transmissibility. The strain with D614G is associated with validated previous studies showing that patients infected have higher viral loads in the upper respiratory tract [4]. With time, the virus can get mutated in various ways, and there should be some interventions that will, in general, improve the prognosis of the disease.

The first line of treatment used in COVID-19 treatment is antiviral drugs with antipyretics etc. As measures to fight the novel COVID-19 are researched, emphasis is being placed on reducing the cytokine storm and immunity-building benefits of COVID-19 treatment.

In the present study, the combination of two tablets (dosage mentioned in the intervention and dosage section), one with Aspirin and Promethazine and the other with vitamin D3, C, and B3 along with zinc and selenium supplementation was proposed as an intervention (APMV2020) (CTRI/2021/06/034254). The ingredients in the formulation represent a precise, tailored therapy for the symptoms of COVID, combined with natural constituents to help the body to build immunity on its own to recover from infection.

Treatment with APMV2020 in mild to moderate COVID 19 management holds the potential for providing faster recovery from associated symptoms and reducing the risk of disease progression [5,6,7]. The present clinical trial involves the evaluation of the safety and efficacy of APMV2020 along with the standard of care in mild to moderate COVID 19 patients. 

Objectives of the study

The objectives of the study were to assess the effectiveness of the APMV2020 combination in reducing symptoms that is more subjects getting relieved of symptoms, reducing inflammatory markers like C-reactive protein (CRP), lactate dehydrogenase (LDH), ferritin, and faster clinical recovery in mild to moderate COVID 19 patients along with the assessment of safety and tolerability of the intervention.

## Materials and methods

Study design

**Figure 1:**
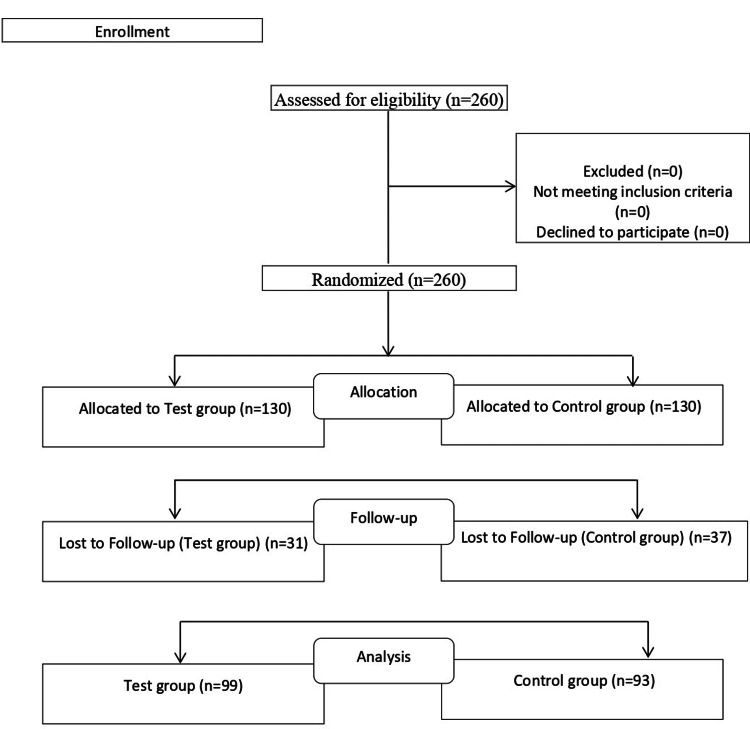
CONSORT diagram.

We conducted a randomized controlled trial with superiority consideration compared to control involving COVID-19 patients recruited from the outpatient department of SK Clinic & Scans, Thiruvananthapuram, Kerala (site one); Swasthi Orthopedic and Respiratory Health Care, Bangalore, Karnataka (site two); Vijay Nursing Home, Nashik Maharashtra (site three). The study was approved by Royal Pune Independent Ethics Committee and was registered with the Clinical Trial Registry of India (CTRI/2021/06/034254). The trial involved parallel design, two groups i.e. APMV2020 and control allocated as 1:1. The consolidated standards of reporting trials (CONSORT) flow of the entire study is depicted as follows (Figure 1).

Inclusion criteria

Mild to moderate symptomatic patients [having National Early Warning Score (NEWS) score ≤ 6] aged 18 to 60 years, males and females, confirmed as COVID-19 positive (maximum of 48 hrs before randomization) based on the everse transcription-polymerase chain reaction (RT-PCR) report were screened for the study. Subjects willing to provide consent and follow-up were included in the study. There were no changes in the inclusion criteria throughout the study.

Exclusion criteria

Patients with autoimmune disease and compromised immunity were not included in the study. Pregnant or lactating women, patients requiring hospital admission at the time of screening, and those with aspirin contraindicated were excluded. Subjects with comorbidity at the critical stage at screening were also excluded.

Study groups

We have taken the patient's consent as per the GCP guidelines. We screened 60 subjects on sites one and two and 140 subjects on site three i.e. Two hundred and sixty subjects based on the inclusion-exclusion criteria, all of which were found suitable and were randomized using a computer-generated randomization sheet to receive either the standard treatment i.e. antiviral and antipyretic agents (control group) or APMV2020 tablets (treatment group). All the subjects were provided with conventional care advocated by the Indian Council of Medical Research (ICMR), Ministry of Health and Family Welfare, Government of India. Figure 1 presents the flow of events for the subjects considered in the analysis for this manuscript. The mechanism used to implement the random allocation sequence was sequentially numbered containers, as the trial is open-label there is no blinding. We received a randomization schedule from a qualified statistician and the investigator enrolled the participants in respective study groups.

Sample size

Considering 40% of subjects getting relieved of cough in the APMV2020 group is on day five compared to 20% in the control group, a qualified statistician evaluated the sample size of a total of 240 (120 cases in each arm) completed cases needed to assess the study objective at 90% power and 5% level of significance. A total of 260 subjects were screened and equally divided in both groups, we represented data of 192 completer subjects in this manuscript. There were 31 subjects from the APMV2020 group and 37 from the control group were lost to follow-up and hence dropped out of the study.

Intervention and dosage

The dose of APMV2020 was tablet A and tablet B one tablet twice a day with water for 10 days (Table 1). The treatment was provided for 10 days.

**Table 1:** APMV2020 details

Component A tablet	
Aspirin IP	150 mg
Promethazine Hydrochloride IP	5 mg
Component B tablet	
Vitamin D3	2000 IU
Vitamin C	750 mg
Niacinamide	80 mg
Zinc Sulphate Monohydrate	15 mg
Potassium Iodide	100 mcg
Sodium Selenate	82.5 mcg

To assure a standard of care treatment antiviral, antipyretic, and antibiotics were used as per the discretion of the investigator to both groups, and additionally, antihistaminic or antiinflammatory agents were prescribed as a rescue to the control group discretion of the investigator wherever needed.

Outcome measures

As APMV2020 contains ingredients such as aspirin and promethazine thought to be reducing symptoms and inflammation in COVID 19 and multivitamin composition aiding early recovery, the primary study outcomes were improvement in clinical symptoms on four points ordinal scale (0- no symptoms, one - mild symptoms, two - moderate symptoms, three - severe symptoms) including fever, headache, diarrhea, breathlessness, cough, anosmia, fatigue, and myalgia, a reduction in elevated levels of inflammatory markers and changes in SpO2 levels. The symptom scoring was performed using WHO eight score ordinal scale. The secondary outcomes were the requirement of hospitalization and admission to ICU, and the adverse events occurring from baseline to the end of the study. There were no changes in the outcome assessment or amendments to the protocol.

Methodology

After attaining ethical approval, the study was registered on the Clinical Trials Registry- India (CTRI) website (CTRI/2021/06/034254). The subjects with positive RT-PCR reports within 48 hrs from the outpatient department of study sites were recruited. The subjects were considered for further evaluation as per the inclusion and exclusion criteria. On the screening visit, written informed consent was obtained from subjects. Demographic details, medical, surgical, and treatment history, and current medication, were noted in the case record form (CRF), along with the vital signs, followed by detailed clinical examination and lab investigations. The record of concomitant medication was properly maintained. The eligible subjects were randomized in respective groups. The treatment was followed till day 10. Assessment of treatment compliance, SpO2, symptom grading was done using the patient’s diary. All subjects were advised to follow their diet routine. The presence of any adverse events was strictly monitored and reported. On day 10, all lab investigations were repeated. The period of the study was from December 2021 to February 2022. There were 95% of subjects fully vaccinated at the time of COVID 19 infection and study enrollment in both groups.

Data analysis

Patients without any major protocol violation like in inclusion criteria and compliance, who consumed at least one dose of intervention, and those who did not take any prohibited medications like any traditional medicine during the study period were considered for analysis. Variables, such as age and gender, were summarized by frequency, mean, and standard deviation. 

Number of subjects presenting score of 0 on days five and 10 were analyzed and compared between groups using the Fisher’s exact test. Levels of inflammatory markers were analyzed by Wilcoxon signed rank test and Mann Whitney test whereas the hematological parameters were assessed by student t-test. Parameters like the requirement of hospitalization and ICU, and days of oxygen supplementation, were represented as percentages.

## Results

Total of 260 subjects were screened and randomized in two groups (130 in each group), out of which 31 subjects from the APMV2020 group and 37 from the control group were dropped out as they lost to follow-up. We evaluated 99 subjects from the APMV2020 group and 93 from control group (total of 192) in this manuscript.

Demographic characteristics

Both groups were comparable in terms of the mean age of the male and female subjects, ranging from 36 to 37.6 years. There were 16 and 18 subjects presenting comorbidity in APMV2020 and control groups respectively. The male to female ratio in both test and control groups was approximately 55.2:44.8 (Table 2).

**Table 2:** Demographic details of study subjects *Analyzed by student t test. # analyzed by chi square test. Non-significant p>0.05

Parameter	Treatment		Control	
Group/ Gender#	Male (n=54)	Female(n=45)	Male(n=52)	Female(n=41)
*Age (years)	38.20±10.52	36.82±10.26	36.27±11.28	36.49±12.67
Total Age (years)	37.6±10.4		36±12	

Primary study outcomes

Change in the COVID-19 Symptoms

Clinical symptoms such as cough, breathlessness, fatigue, myalgia, headache, diarrhea and anosmia were assessed from baseline to day 10. There was significant reduction (p<0.05) in symptoms in both groups from their respective baseline to day 10. However it was evident that the treatment group had a faster relief of symptoms compared to the control as more subjects were relieved of cough, myalgia, headache and anosmia in the treatment group on day five as compared to the control (Table 3). The faster resolution of symptoms is denoted by change of WHO ordinal scale score from two to 0 on day five in the treatment group than the control.

**Table 3:** Subjects population with symptom score between groups Analyzed by Fisher Exact test. p<0.05 indicated as * for within group and # for between group analysis.

Duration/ score	Treatment (N=99)			Control (N=93)		
	Score 0	Score 1	Score 2	Score 0	Score 1	Score 2
Cough						
Baseline	39	26	34	46	12	35
5	56*	37	6	49	34	10
10	62*	37	0	55	38	0
Breathlessness						
Baseline	97	2	0	92	1	0
5	99	0	0	90	1	2
10	99#	0	0	89	2	2
Fatigue						
Baseline	95	2	2	91	2	0
5	98	1	0	88	1	4
10	99#	0	0	85*	1	7
Myalgia						
Baseline	34	35	30	29	30	34
5	77*#	21	1	59*	33	1
10	97*	2	0	93*	0	0
Headache						
Baseline	57#	26	16	67	17	9
5	86*	13	0	87*	4	2
10	98*	1	0	93*	0	0
Diarrhea						
Baseline	91	3	5	88	3	2
5	99*	0	0	93*	0	0
10	99*	0	0	93*	0	0
Anosmia						
Baseline	83	6	10	80	6	7
5	97*#	2	0	81	10	2
10	99*#	0	0	83	9	1

Changes in SpO2 Levels

The average SpO2 level in both groups were maintained in normal range and thus the difference was not significant (The range of SpO2 was 97-98.5%).

Changes in Inflammatory Markers

Inflammatory markers, namely CRP, LDH, D-dimer, and ferritin were elevated in both groups at baseline. There was a statistically significant (p<0.05) reduction in the elevated levels of serum LDH and ferritin in the treatment group compared to the control (Table 4). There was a statistically significant reduction (p<0.001) in both groups but more reduction in magnitudes of the treatment group.

**Table 4:** Changes in inflammatory markers between groups Data analyzed by-Within group comparison: Wilcoxon signed-rank test (CRP), student t-test (LDH and ferritin. Between-group comparison: Maan Whitney U test (CRP), student t-test (LDH and ferritin)

(Mean ± SD) LDH IU/L			
Duration	Treatment (N=99)	Control (N=93)	P value (Between)
Screening	363.77±126.54	347.93±136.26	0.406
Day 10	296.61±102.59	330.46±122.15	
Mean diff (Screening – Day 10)	66.32±111.85	17.47±106.04	0.002
% Reduction	18.42%	5.02%	
P value (within)	<0.001	0.115	
(Mean ± SD) Ferritin ug/L			
Duration	Treatment (N=99)	Control (N=93)	P value
Screening	106.38±88.23	105.55±98.73	0.931
Day 10	87.41±77.55	104.62±113.42	
Mean diff (Screening – Day 10)	18.97±59.74	0.59±80.96	0.074
% Reduction	17.83%	0.88%	
P value (within)	0.002	0.944	
(Mean ± SD) CRP mg/L			
Duration	Treatment (N=99)	Control (N=93)	P value
Screening	9.21±13.66	9.40±7.59	0.327
Day 10	3.95±2.83	4.73±2.59	
Mean diff (Screening – Day 10)	5.26±13.62	4.52±7.48	0.772
% Reduction	57.11%	49.63%	
P value (within)	<0.001	<0.001	

Secondary study outcomes

Hospitalization, ICU, and Supplemental Oxygen

There were no subjects who required hospitalization, ICU, and supplemental oxygen till their clinical recovery in both groups.

Safety outcomes

Hematological parameters were assessed on baseline and day 10, and there were no significant changes in the parameters between groups (Table 5).

**Table 5:** Changes in hematological parameters between groups Analyzed by student t-test. Not significant (p>0.05).

Laboratory Investigation	(Mean ± SD)			
	Test		Control	
	Baseline	Day 10	Baseline	Day 10
Total Leukocyte Count (/mm^3)	7743.94±2294.34	7440.91±1830.71	7907.10±2664.26	7281.18±1764.29
Neutrophil %	58.34±9.27	56.76±7.03	58.77±10.47	56.14±7.73
Lymphocyte %	36.12±9.04	36.49±6.42	35.17±10.69	37.33±7.90
Monocyte %	3.25±1.21	3.74±1.75	3.43±1.32	3.68±1.70
Eosinophil %	3.45±2.31	3.57±2.12	3.26±1.99	3.43±1.98
Total RBC 10^6/mm^3	4.76±0.70	4.82±0.55	4.67±0.69	4.76±0.51
Hemoglobin (g/dl)	13.14±1.90	13.14±1.74	12.97±1.73	12.97±1.61
Hematocrit (%)	41.11±4.79	41.42±4.25	40.35±5.31	40.61±4.40
Platelet Count (Lacs/mm^3)	2.72±0.74	3.02±0.67	2.57±0.75	2.76±0.64

Adverse events

No adverse events related to study medication or possible engagement of test intervention were reported throughout the study period.

## Discussion

This randomized controlled trial evaluated the effects of the APMV2020 tablets on COVID-19. Patients of the APMV2020 group demonstrated faster recovery from symptoms of COVID-19 evident by more and more subjects getting relieved of symptoms in five days, such as cough, myalgia, headache, and anosmia, and reduced serum levels of inflammatory markers like LDH, ferritin, and CRP. Overall, the inclusion of APMV2020 in the treatment protocol for COVID-19 resulted in faster clinical recovery in terms of symptomatic relief with no disease progression. The duration of the study medication was of 10 days. We followed up with all subjects for 28 days telephonically on days 15, 20, and 28 to assess their wellness. It was found that all subjects in both groups were clinically recovered and did not present any major symptoms.

Repurposing the use of acetylsalicylic acid (aspirin) is a reasonable choice to come up to a sustainable intervention in mild, moderate to severe patients with COVID 19. Aspirin is a researched molecule for its anti-inflammatory, analgesic, and antithrombotic properties. It has antiviral potential of its effect against DNA and RNA viruses. Aspirin was reported to be reducing RNA synthesis and replication of human coronavirus-299E (CoV-229E) and Middle East Respiratory Syndrome (MERS)-CoV in an in vitro study [5].

Remarkably, the use of aspirin in COVID-19 is explored for its possible causal effect in reducing mortality, hospital stay, and supplemental oxygen requirement. As per research by Chow et al. the use of aspirin was associated with improved outcomes among hospitalized patients with COVID-19 [6].

Patients with COVID-19 have positively correlated with systemic elevation of pro-inflammatory cytokines IL-6 and TNF-α [6]. The over-activation of mast cells and release of cytokines might also have a role in the development of pulmonary fibrosis in COVID-19. An anti-histaminic agent like Promethazine can act in COVID-19 patients as a cough suppressant as well as a strong anti-inflammatory agent to reduce histamine and mast cell activity [7].

The multivitamin combination of APMV2020 can be beneficial in supporting the immune system as well as it’s a unique blend of Vitamin D3, C, and B3 along with zinc, thereby having the potential to modulate immunity to achieve faster viral clearance as well as the reduction in extrapulmonary effects of the infection to lead to speedy clinical recovery.

It is well documented through previous research that zinc has the potential to inhibit viral replication. A wide range of viruses is reported to be inhibited in vitro using zinc including SARS coronavirus (SARS-CoV). In the later stages of infection, zinc could be beneficial by mitigating the impact of deregulation of the immune system, inflammation, and hypoxia‐induced oxidative stress by modulating the immune response, and inflammatory pathway it improves recovery of patients in viral infection. Zinc can target multiple pathways driving the complex pathogenesis of COVID-19 and thus would be beneficial in COVID-19 management [8].

In the present research, there was no need of hospital admission, nor supplemental oxygen in both groups. 

Assessment of hematological parameters suggested no significant change post-treatment and no adverse events throughout the 10-day protocol, indicating the safety of the intervention. All patients were compliant with the APMV2020 regimen for 10 days. Symptom regressions and recovery are strongly correlated in COVID 19 [9]. Our results demonstrated that APMV2020 significantly relieved symptoms such as cough, myalgia, headache and anosmia for more subjects faster than the control group.

Triggered inflammatory responses result from rapid viral replication of SARS-CoV-2 or other inflammatory conditions, resulting in cellular destruction that can stimulate the release of cytokines and chemokine through macrophages and monocytes leading to cytokine storms in COVID-19. Inflammatory markers, such as serum LDH, ferritin and CRP have been positively allied to the high risks of severity and fatality in COVID-19 [10].

In the present study, treatment with APMV2020 demonstrated excellent anti-inflammatory activity by significantly reducing serum levels of LDH, ferritin, and CRP than control. APMV2020 is a potential interventional candidate with balanced immunomodulatory and anti-inflammatory activities.

Aspirin from APMV2020 inhibits platelet aggregation and cyclooxygenase activity causing inhibition of Thromboxane-A2, which is responsible for inflammation and thrombosis which accounts for pulmonary and cardio-protective benefits of Aspirin. Aspirin can help in the prevention of thrombo-inflammation, pulmonary embolism, and thrombosis found commonly in COVID-19 patients [11]. There are many studies that provide data regarding the usefulness of vitamin D3, B3 and C in COVID 19 management by virtue of their powerful antioxidant, anti-inflammatory and immunomodulatory activities [12].

Selenoproteins have a crucial role in maintaining redox balance virtually in all tissues [13]. COVID-19 is characterized by increased oxidative stress; therefore, selenium is thought to be beneficial in the management of COVID-19. A study demonstrated the use of selenium in subjects to achieve rapid clinical recovery in mild to moderate COVID-19 patients to reduce mortality and hospital stay in severe COVID-19 [13].

The unbalance nutrition affects the immune system and can induce inflammatory cascade, which is prevalently seen in severe infection with SARS-CoV-2. The severity in COVID-19 is seen in older people, comorbid, malnourished and obese individuals. There are many studies suggesting a role of vitamins and minerals like vitamin A, D, zinc and selenium in alleviating inflammatory response in COVID-19 [14].

Nutritional supplementation correcting nutritional imbalance is important in promoting a diverse gut microbiota thus supporting the optimum immune response to any infection. There are many advantages of a balanced nutritional therapy like the APMV202 relevant to encounter with SARS-CoV-2 infections [15].At present, there are no elaborative studies to confirm that micronutrient deficiency just like selenium can trigger the mutations in viral RNA like in influenza and coronaviruses [16].

To summarize, APMV2020 can prove a good candidate to be integrated into the COVID-19 management protocol as it aids in faster symptom recovery with significant anti-inflammatory activity and has potential immunomodulatory properties by virtue of the multivitamin combination of the APMV2020. A clinical trial of APMV2020 is warranted in long COVID patients.

## Conclusions

It can be concluded that APMV2020 treatment for COVID-19 patients provides advantages over the standard of care treatment alone. There was a faster recovery of subjects from COVID-19 symptoms, along with a significant reduction in inflammatory markers like LDH ferritin, and CRP. This study serves as preliminary evidence for further research using aspirin, promethazine, vitamin D, C, and micronutrient therapy as an intervention in the management of long COVID symptoms.
