# Correction: Efficacy and Safety of Aspirin, Promethazine, and Micronutrients for Rapid Clinical Recovery in Mild to Moderate COVID-19 Patients: A Randomized Controlled Clinical Trial

**DOI:** 10.7759/cureus.c66

**Published:** 2022-06-13

**Authors:** G. Sunil Kumar, Atul Vadgaonkar, Srilata Purunaik, Rohit Shelatkar, Vidyadhar G Vaidya, Gayatri Ganu, Aditya Vadgaonkar, Shashank Joshi

**Affiliations:** 1 Cardiology, S K Clinic & Scans, Thiruvanathapuram, IND; 2 General Medicine, Vijay Nursing Home, Nashik, IND; 3 Respiratory Medicine, Swasthi Orthopaedic and Respiratory Health Care, Bengaluru, IND; 4 Pharmacology and Therapeutics, Vitabiotics Ltd., London, GBR; 5 General Surgery, Lokmanya Medical Research Centre, Pune, IND; 6 Pharmacology and Therapeutics, Mprex Healthcare Pvt. Ltd., Pune, IND; 7 Medicine, King Edward Memorial Hospital and Seth Gordhandas Sunderdas Medical College, Mumbai, IND; 8 Endocrinology, Lilavati Hospital, Mumbai, IND

This article has been corrected to add a financial conflict of interest that was not previously disclosed at the time of submission and publication.

The fourth author, Rohit Shelatkar, is employed by Vitabiotics Ltd. working in departments for new product development, clinical trials, and consumer trials. The study product in this article was manufactured by the study sponsor, Meyer Organics Pvt. Ltd. Meyer Organics Pvt. Ltd is part of Vitabiotics Ltd. While Rohit Shelatkar's affiliation was correctly included, this connection between the study sponsor, Meyer Organics Pvt. Ltd, and Dr. Shelatkar's employer, Vitabiotics Ltd. should have been clearly disclosed. The authors sincerely regret this error of omission.

